# Liver Tumor Enhancement at Hybrid Angio-CT and Comparison With Tumor and Hepatic Parenchymal Distribution of Yttrium-90 Microspheres by Positron Emission Tomography

**DOI:** 10.7759/cureus.49861

**Published:** 2023-12-03

**Authors:** Garth S Campbell, Dustin K Reed, Ajinkya Desai, Seth T. Lirette

**Affiliations:** 1 Interventional Radiology, University of Mississippi Medical Center, Jackson, USA; 2 Interventional Radiology, Mississippi Baptist Medical Center, Jackson, USA; 3 Data Science, University of Mississippi Medical Center, Jackson, USA

**Keywords:** liver metastases, hepatocellular carcinoma, angiography, interventional radiology, positron emission tomography, radiation microsphere therapy, selective internal radiotherapy, radioembolization, yttrium-90

## Abstract

This single-center retrospective study evaluated patients who underwent treatment of a primary or secondary hepatic malignancy with injection of glass or resin yttrium-90 (^90^Y) microspheres with a corresponding hybrid angiography-computed tomography (angio-CT) and ^90^Y positron emission tomography (PET). Volumetric contours were defined by three independent observers and were used to calculate relative tumoral enhancement at angio-CT. This parameter was compared with the tumor-to-normal (T/N) activity ratio predicted by technetium-99m macro-aggregated albumin (^99m^Tc-MAA) single photon emission computed tomography (SPECT) and microsphere activity distribution by ^90^Y PET. A similar correlation was observed for the enhancement ratio at angio-CT with observed microsphere distribution at ^90^Y PET (r=0.34) to that predicted by ^99m^Tc-MAA SPECT (r=0.32). The enhancement ratio on angio-CT performed as well as ^99m^Tc-MAA in the prediction of ^90^Y PET activity distribution. The technique could not be readily applied to tumors with large areas of hypoattenuation (necrosis) on angio-CT. With refinement and further study, this technique could be used as a quantitative adjunct to standard-of-care ^99m^Tc-MAA SPECT for dosimetry calculations and prediction of microsphere distribution to maximize tumor response and minimize hepatotoxicity.

## Introduction

Treatment of hepatic malignancy with radioactive microspheres, known alternatively as trans-arterial radioembolization (TARE), selective internal radiation therapy (SIRT), or radiation microsphere therapy (RMT), is accomplished by selective arterial infusion of the microspheres which are labeled with a beta particle-emitting radioisotope [1]. Tumoral and hepatic parenchymal radiation doses are dependent not only on the activity selected for infusion, but also on the microsphere distribution, optimization of which has led to improved tumor response and clinical outcomes through radiation segmentectomy and personalized dosimetry [2,3]. Dosimetry for either paradigm is dependent on accurate volume calculation, and usage of the personalized dosimetry concept requires the prediction of microsphere deposition by modeling a presumably similar distribution of technetium-99m macro-aggregated albumin (^99m^Tc-MAA) injected from the planned point of microsphere infusion with gamma camera or single-photon emission computed tomography (SPECT). The relative distribution of activity is represented by the tumor-to-tumor uptake ratio or T/N ratio (TNR) [4]. Techniques for non-invasive measurement of the in-vivo activity distribution by yttrium-90 (^90^Y) positron emission tomography (PET) have previously been described and characterized [5,6].

Preoperative contrast-enhanced diagnostic imaging with computed tomography (CT) or magnetic resonance imaging (MRI) provides information about liver and tumor volume and offers lesion enhancement as a proxy for lesion vascularity but does not provide the explicit information needed for calculation of perfused volume or TNR for microsphere dosimetry, both of which depend on the infusing microcatheter position. Hybrid angiography-CT (angio-CT), in which a traditional fluoroscopic angiography suite is integrated with an in-room CT scanner, allows infusion of radiopaque contrast through a microcatheter in the planned position for microsphere administration. This technique has previously been used to assist in delineation of vascular anatomy, provide accurate volume calculations for dosimetry, and improve lesion detection over conventional C-arm cone-beam CT [7]. Angio-CT provides greater resolution and real-time information to the interventionalist to assist in treatment decisions and dosimetry calculations.

The purpose of this study was to evaluate and compare the enhancement observed at angio-CT to the TNR predicted by ^99m^Tc-MAA SPECT and the actual microsphere distribution observed on ^90^Y PET by independent observers for both glass and resin 90Y microspheres. 

## Materials and methods

From September 2020 to September 2021, 56 dose distribution ^90^Y PET scans were performed for patients undergoing intra-arterial 90Y microsphere treatment. Patients with a corresponding angio-CT were selected for analysis. Patients who were treated with a microsphere infusion in different arteries on the same day were excluded from the analysis. One patient was excluded from the analysis due to corrupted PET data.

Institutional review board (IRB) approval was obtained for this retrospective analysis, and patient anonymity was maintained throughout the study. A waiver of informed consent for this retrospective review was provided by the IRB. Informed consent was obtained and documented from all patients for clinical treatment. At the time of this study, glass microsphere treatments were performed under a humanitarian device exemption (HDE) supervised by the IRB, and patients signed an additional IRB-approved informed consent form for those treatments.

Procedure and imaging

All planning angiography and microsphere infusions were performed by one of three interventional radiologists with 6, 10, or 11 years of experience. Patients were selected for treatment after review at a multispecialty tumor board. After consultation and informed consent, all patients underwent planning arteriography in a dedicated angiography suite with an integrated CT scanner (Siemens Healthineers, Erlangen, Germany). ^99m^Tc-MAA injection was performed during planning arteriography in the artery intended for microsphere infusion. Planar and SPECT images were obtained immediately following planning arteriography on Infinia (GE Healthcare, Chicago, IL) or Symbia Evo (Siemens Healthineers, Erlangen, Germany) gamma camera systems, with estimation of lung shunt fraction from the planar images. 

Dosimetry was performed by the treating interventional radiologist in collaboration with a medical physicist, and the written directive for each treatment was reviewed. The microsphere type, nominal vial activity at calibration, total injected activity, and estimated lung shunt fraction were recorded from the written directive.

Microsphere administration was performed a median of 16 days after planning arteriography, with placement of a microcatheter into the target artery and microsphere infusion by the interventional radiologist. The date and time of microsphere infusion were recorded for decay calculations. PET/CT dose distribution scans were performed on a 64-slice Discovery 690 PET/CT (GE Healthcare, Chicago, IL) with the apparent isotope for detection set to either fluorine-18 (^18^F) or sodium-22 (^22^Na). The date and time of the PET/CT scan were also recorded for decay calculations. 

Angio-CT images were performed at the discretion of the treating interventional radiologist during planning arteriography, microsphere administration, or both. Images were obtained on a 64-slice CT scanner in a Nexaris hybrid angio-CT suite (Siemens Healthineers, Erlangen, Germany). Omnipaque 300 contrast (GE Healthcare, Chicago, IL) was diluted to 30% concentration with normal saline and infused with a power injector (Guerbet, Villepinte, France) in the hepatic arterial location and at a rate selected by the treating interventional radiologist based on flow observed at angiography. The delay between contrast injection and CT scanning was not recorded for each patient, but a departmental protocol was adopted during the study period that fixed the delay at 8 seconds unless the operator specified otherwise. Contrast was continually injected into the artery throughout the duration of the delay and the period of CT scanning.

Imaging analysis and calculation of tumor-to-normal ratio and enhancement

For each case, the following image series were de-identified and imported into MIM software (MIM Software Inc, Beachwood, OH): attenuation-corrected time-of-flight (TOF) PET images and associated CT slices, angio-CT images, and SPECT images. When more than one set of angio-CT images were available, the series most closely resembling the microcatheter position for microsphere infusion was chosen.

**Figure 1:**
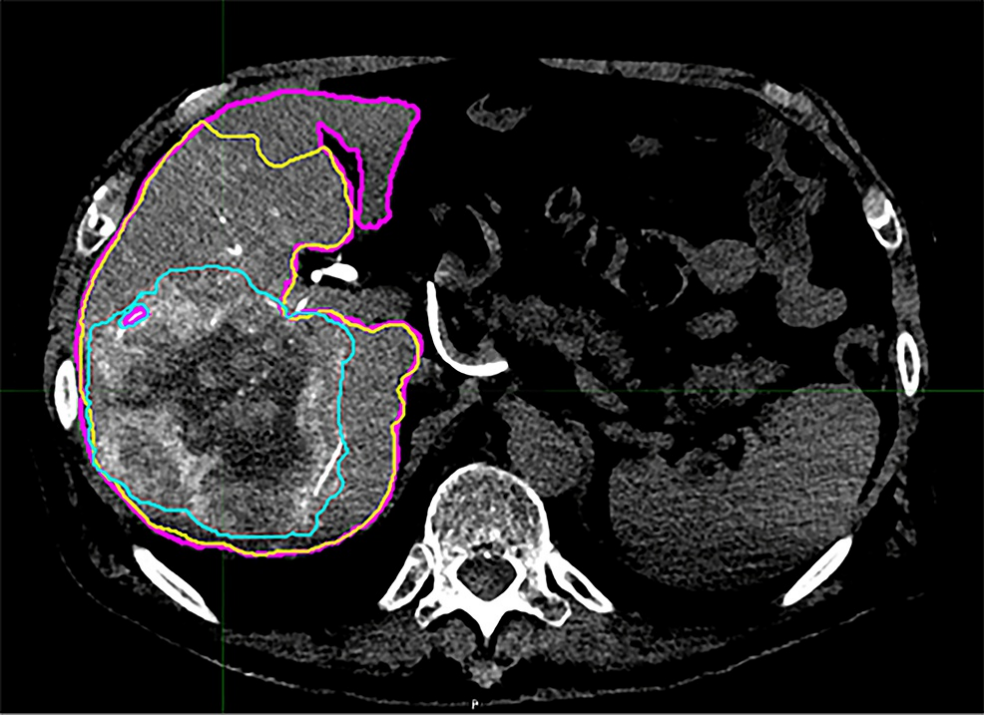
Representative cross-section of operator-defined contours defined on intraprocedural angio-CT Shown are the total liver (magenta), perfused territory (yellow), and tumor (cyan) contours.  The nontumor perfused and unperfused contours are derived from these operator-defined contours by Boolean operation in the software.

**Figure 2:**
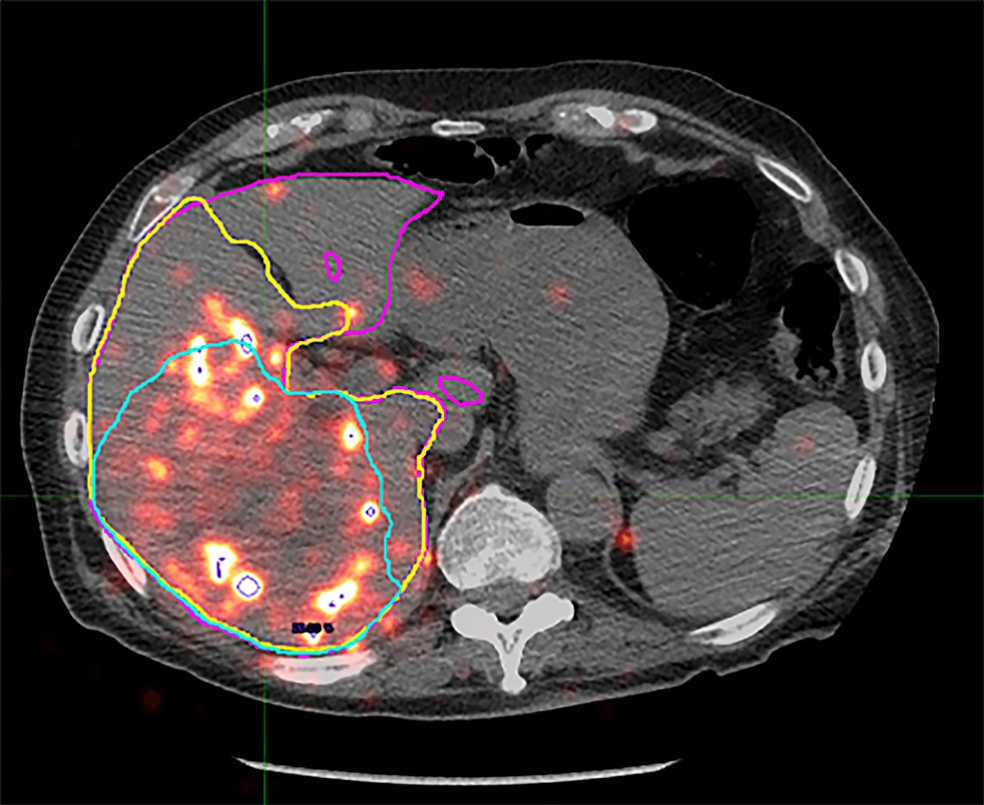
Representative cross-section of operator-defined contours projected on fused 90Y PET/CT Shown are the total liver (magenta), perfused territory (yellow), and tumor (cyan) contours.  The nontumor perfused and unperfused contours are derived from these operator-defined contours by Boolean operation in the software.

Contour volumes were defined in the MIM software by each of the three independent observers based on the angio-CT images imported. Observers were not provided with preprocedural diagnostic imaging for comparison but followed the same set of directions in producing the contours. All contours were drawn on the angio-CT images and then transferred to the other series. Contour volumes were defined for the liver, perfused volume, and tumor. Contours were then derived for the part of the liver that was not perfused (“unperfused liver”) and the normal parenchyma in the perfused liver (“nontumor perfused”) by a Boolean operation function in the software (i.e., unperfused = total liver - perfused; nontumor perfused = perfused - tumor). Variables recorded for each defined contour included the contour volume by angio-CT, integral activity detected by PET, and integral counts detected by SPECT. Representative images of the operator-defined contour volumes fused to the PET and SPECT images are shown in Figure 1 and Figure 2. 

Calculation of the tumor-to-normal ratio for the dose-distribution PET (PTNR) was obtained by dividing the average activity within the tumor volume by the average activity in the nontumor perfused volume (Equation 1). Similarly, calculation of the tumor-to-normal ratio prediction by SPECT (STNR) was obtained by dividing the average counts within the tumor volume by the average counts in the nontumor perfused volume (Equation 2).

Equation 1

$PTNR= \frac{\frac{Tumor\, Intergral\, Activity}{Tumor\, Volume}}{\frac{Nontumor\, Perfused\, Integral\, Activity}{Nontumor\, Perfused\, Volume}}$ 

*Equation 2* 

$STNR= \frac{\frac{Tumor\, Intergral\, Counts}{Tumor\, Volume}}{\frac{Nontumor\, Perfused\, Integral\, Counts}{Nontumor\, Perfused\, Volume}}$

 An “enhancement ratio” (ENR) was defined as the enhancement of the tumor contour divided by the enhancement of the nontumor perfused contour (Equation 3). Because an unenhanced CT scan was not routinely obtained prior to the angio-CT, the average attenuation of the unperfused liver contour was used as a surrogate for unenhanced attenuation. The “enhancement” of each contour was then defined as the average attenuation in Hounsfield Units (HU) of that contour minus the average attenuation of the unperfused liver contour.

Equation 3

$ENR= \frac{Tumor\, Mean\, Attenuation-Unperfused\, Mean\, Attenuation}{Nontumor\, Perfused\, Mean\, Attenuation-Unperfused\, Mean\, Attenuation}$

​​​​Statistical analysis

Study data were collected and managed using REDCap data capture software. Summary statistics were compiled where appropriate and compared with t-test or Fisher’s exact tests. Scatterplots were produced for ENR, STNR, and PTNR against one another and the correlation coefficient was calculated for each comparison of glass and resin microspheres separately and combined. The intraclass correlation coefficient (ICC) was calculated from random effects models and was paired with corresponding Bland-Altman plots for each of these variables to assess observer agreement. All statistical analyses were performed with Stata v17.1 (StataCorp LLC, College Station, Texas, USA).

## Results

Baseline characteristics of treatments

Of the 43 treatments included in the study, 33 were performed for hepatocellular carcinoma using glass microspheres and 10 were performed for other malignancies in the liver (one intrahepatic cholangiocarcinoma, seven colorectal carcinomas, and two neuroendocrine tumors) using resin microspheres. Glass microsphere infusions were more commonly performed at the sublobar or segmental level, while resin microsphere infusions were more commonly performed at the lobar level. The mean injected activity was higher for glass (2.24 ± 1.19 GBq) compared to resin (1.29 ± 0.7 GBq) microspheres, and the mean number of particles infused was lower for glass (3.85 ± 3.57 x 106) compared to resin (19.2 ± 10.3 x 106) microspheres. The baseline characteristics of patients treated with glass and resin microspheres are shown in Table 1.

**Table 1:** Baseline characteristics of treatments

Microsphere Type	Glass	Resin	p-value
Hepatocellular carcinoma	33	0	<0.001
Other Malignancy	0	10	<0.001
Lobar Infusion	12	6	<0.001
Sublobar or Segmental Infusion	21	4	
Lung Shunt Fraction (%)	8.2 ± 5	5.75 ± 1.9	0.052
Total Injected ^90^Y Activity (GBq)	2.24 ± 1.19	1.29 ± 0.7	0.004
Injected Particles (1x10^6^)	3.85 ± 3.57	19.2 ± 10.3	<0.001

Comparison of the enhancement ratio with SPECT and PET/CT activity distribution

**Figure 3:**
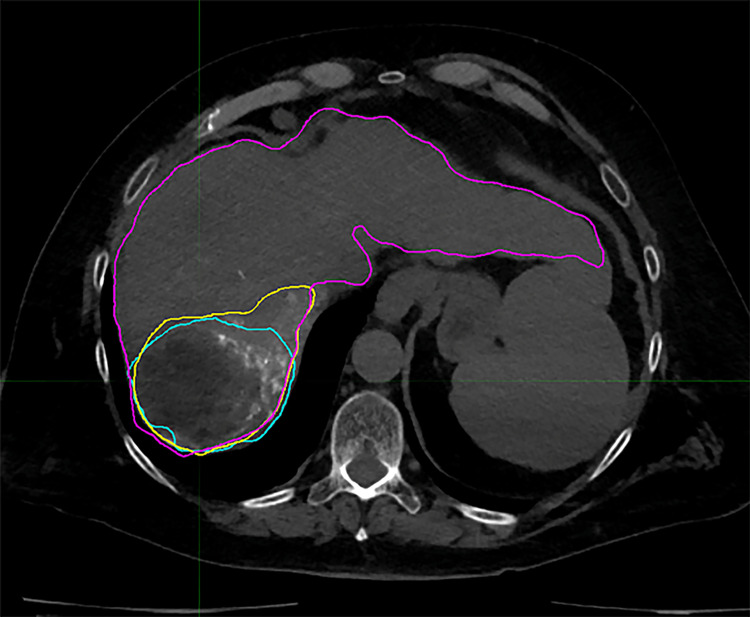
Representative cross-section from operator-defined contours in a patient with a tumor featuring a large area of low-attenuation (necrosis) Shown are the total liver (magenta), perfused territory (yellow), and tumor (cyan) contours.  The tumor contour contains a large nonenhancing and low-attenuation area.

The mean ENR for HCC was 3.38 ± 2.73, and the mean ENR for the other hepatic malignancy was 1.70 ± 1.52 (p=0.258). Negative ENR values were obtained for a small number of measurements; this occurred consistently for all three observers in three instances and for a single observer in two instances. These measurements were excluded from the scatterplots and analysis since the concept of “negative enhancement” was an unintended artifact of the calculation. This was caused by the use of the unperfused liver as a surrogate for an unenhanced scan, occasionally resulting in a higher attenuation for the unperfused liver than the perfused liver. A post hoc review of the images showed that in all three cases where the observers agreed on a negative ENR, the tumors contained large areas of low attenuation/necrosis compared to the high attenuation/enhancing portion. A representative image of a low-attenuation tumor with operator contours is shown in Figure 3.

The mean STNR for HCC was 1.98 ± 1.12, and the mean STNR for the other hepatic malignancy was 2.27 ± 2.19 (p=0.931). The mean PTNR for HCC treated with glass microspheres was 2.75 ± 2.66, and the mean PTNR for other hepatic malignancy treated with resin microspheres was 1.60 ± 0.91 (p=0.090). The mean values of ENR, STNR, and PTNR obtained for HCC and other hepatic malignancies are summarized in Table 2.

**Table 2:** Enhancement ratio and T/N ratios of hepatocellular carcinoma and other hepatic malignancies T/N: Tumor to normal; HCC: hepatocellular carcinoma; ENR: enhancement ratio; STNR: tumor-to-normal activity ratio predicted by SPECT; PTNR: tumor-to-normal activity ratio observed on ^90^Y PET

Type of Malignancy	HCC	Other Hepatic Malignancy	p-value
ENR	3.38 ± 2.73	1.70 ± 1.52	0.258
STNR	1.98 ± 1.12	2.27 ± 2.19	0.931
PTNR	2.75 ± 2.66	1.60 ± 0.91	0.090

**Figure 4:**
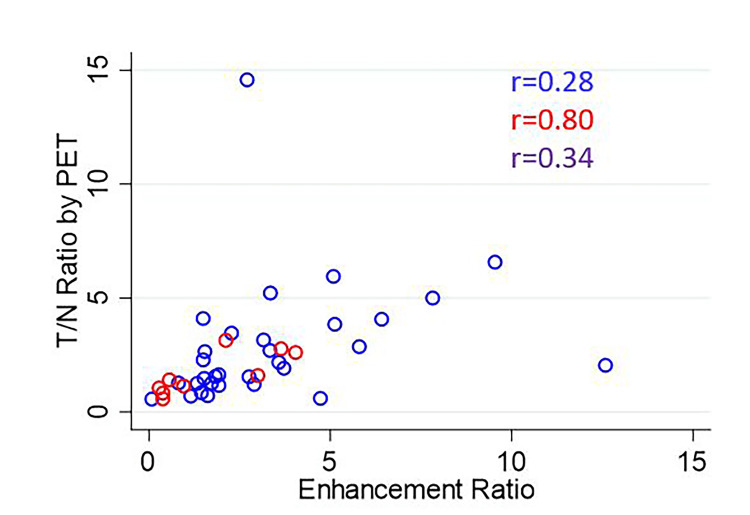
Scatterplot of ENR vs. PTNR The correlation coefficient is listed for each comparison for glass microspheres (blue), resin microspheres (red), and overall (purple). ENR: Enhancement ratio; PTNR: tumor-to-normal activity ratio observed on ^90^Y PET

**Figure 5:**
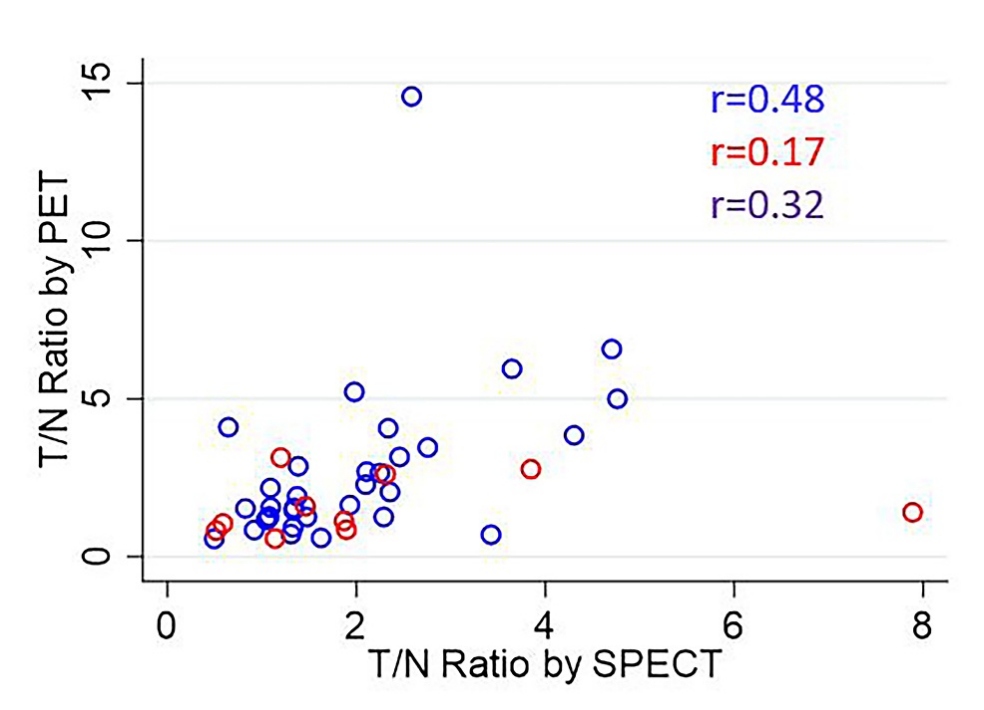
Scatterplot of STNR vs. PTNR The correlation coefficient is listed for each comparison for glass microspheres (blue), resin microspheres (red), and overall (purple). STNR:  Tumor-to-normal activity ratio predicted by SPECT; PTNR: tumor-to-normal activity ratio observed on ^90^Y PET

**Figure 6:**
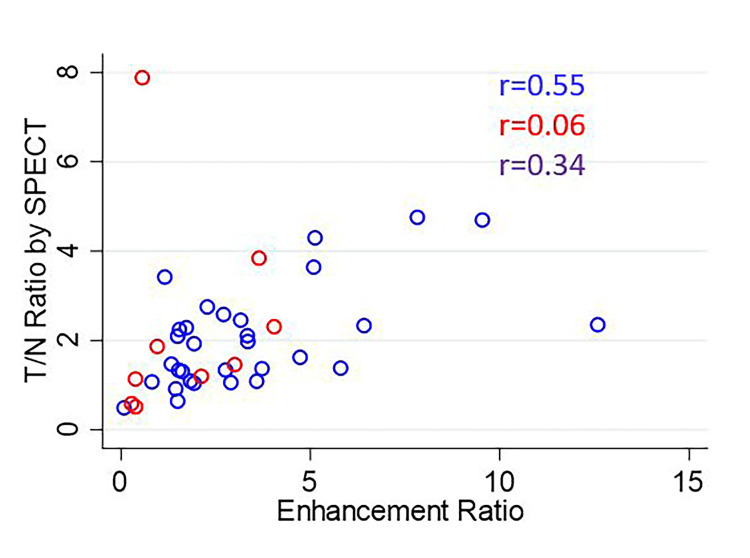
Scatterplot of ENR vs. STNR The correlation coefficient is listed for each comparison for glass microspheres (blue), resin microspheres (red), and overall (purple). ENR: Enhancement ratio; STNR: tumor-to-normal activity ratio predicted by SPECT

The overall correlation coefficients for ENR vs. PTNR, STNR vs. PTNR, and ENR vs. STNR were 0.34, 0.32, and 0.34, respectively. The scatterplot of ENR vs. PTNR is shown in Figure 4. The scatterplot of STNR vs. PTNR is shown in Figure 5. The scatterplot of ENR vs. STNR is shown in Figure 6.

**Figure 7:**
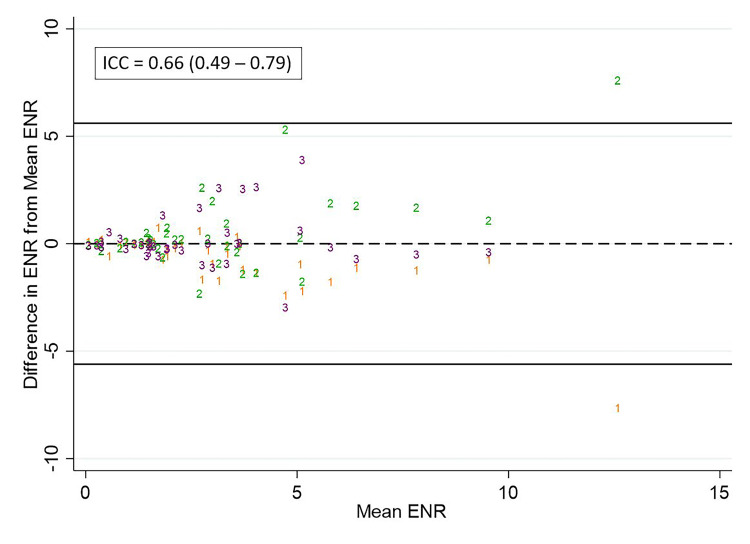
Differences in observer measurements of ENR and calculated ICC Differences in ENR from the mean for each measurement are plotted against the mean for observer 1 (orange), observer 2 (green), and observer 3 (purple).  The calculated intraclass correlation coefficient (ICC) is listed above. ENR: Enhancement ratio

**Figure 8:**
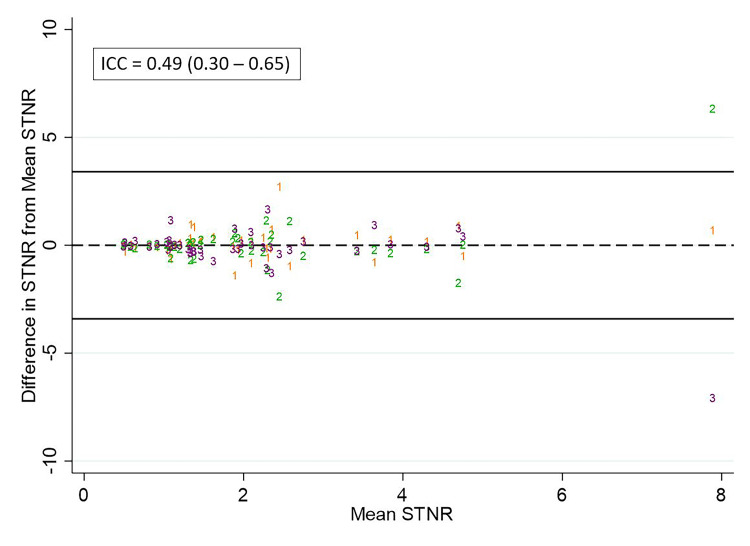
Differences in observer measurements of STNR and calculated ICC Differences in STNR from the mean for each measurement are plotted against the mean for observer 1 (orange), observer 2 (green), and observer 3 (purple).  The calculated intraclass correlation coefficient (ICC) is listed above. STNR: Tumor-to-normal activity ratio predicted by SPECT

**Figure 9:**
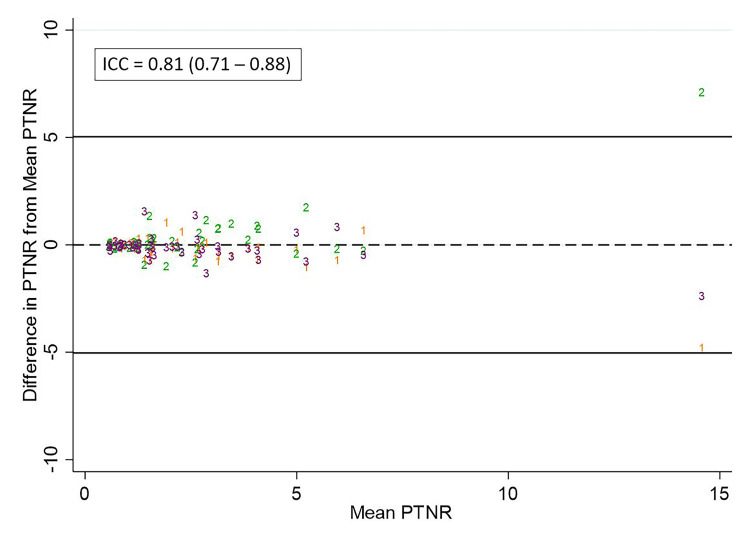
Differences in observer measurements of PTNR and calculated ICC Differences in PTNR from the mean for each measurement are plotted against the mean for observer 1 (orange), observer 2 (green), and observer 3 (purple).  The calculated intraclass correlation coefficient (ICC) is listed above. PTNR: Tumor-to-normal activity ratio observed on ^90^Y PET

The ICC for ENR, STNR, and PTNR measurements was 0.66, 0.49, and 0.81, respectively. Scatterplots of the difference of observer measurements from the mean for ENR, STNR, and PTNR are shown in Figure 7, Figure 8, and Figure 9, respectively. 

## Discussion

This single-center, multiple-observer, retrospective study demonstrates similar correlation for the ENR with observed activity distribution at ^90^Y PET to that for ^99m^Tc-MAA SPECT. 

Excellent correlation was previously reported between the T/N ratio and predicted radiation dose obtained by planar ^99m^Tc-MAA scintigraphy and those obtained from an intraoperative beta probe at laparotomy (r=0.82 for T/N ratio) [4,8]. Subsequent studies evaluating the correlation of ^99m^Tc-MAA SPECT with Bremsstrahlung ^90^Y SPECT have reported lower agreement between these modalities. Wondergem et al. found an over- or underestimation of >30% activity between ^99m^Tc-MAA SPECT and ^90^Y bremsstrahlung SPECT in at least one segment of 82% of procedures, while Ilhan et al. found a weak but significant correlation between ^99m^Tc-MAA SPECT and ^90^Y bremsstrahlung SPECT “tumor to background” uptake (r=0.26) [9,10]. 

^90^Y PET may be the noninvasive “gold standard” for microsphere distribution; an animal study of rabbit VX2 liver tumors found excellent correlation between tumor activity observed at ^90^Y PET and corresponding ex vivo quantification of tissue aluminum content (a constituent of the ceramic microsphere structure) by inductively coupled plasma emission spectroscopy (r= 0.896) [11]. Kafrouni et al. found a close relationship between the predicted dose based on normalized ^99m^Tc-MAA SPECT and that observed at ^90^Y PET for tumor and normal liver (r=0.87 and r=0.91, respectively) [12]. Song et al. also found good agreement between ^99m^Tc-MAA SPECT and ^90^Y PET for the tumor and “in-target normal” liver (the equivalent of “non-tumor perfused” in our study) with r=0.64 and r=0.71, respectively [13].

Although the ICC measurements in our study indicated moderate to good agreement between observers, the variation in perfused and tumor volume measurements between observers may relate to the fact that observers were not privy to additional clarifying preoperative or postoperative diagnostic imaging. Additionally, no limits on the size or number of lesions were imposed on the observers in defining the tumor volumes. The QUEST phantom study showed a steady decline in activity recovery for ^90^Y phantom spheres below 37 mm diameter due to partial volume effects [14]. 

The weaknesses of this study include its retrospective design and the small number of patients, particularly those treated with resin microspheres and hepatic malignancies other than HCC. Discrepancies encountered between some of the observer measurements suggest that some context in the form of preoperative diagnostic imaging or personal knowledge of catheter infusing position may have been helpful. Initially, inconsistent use and lack of standardization of the angio-CT protocol may also have contributed to this uncertainty. Lastly, the use of the ENR as a predictor for activity distribution was limited by the lack of a true unenhanced CT, and the use of the unperfused liver compartment attenuation as a surrogate was not satisfactory when tumors contained large areas of necrosis.

The use of a true unenhanced CT in future studies would allow analysis of the ENR for necrotic tumors and other scenarios where the tumor baseline attenuation may not closely match that of the normal liver parenchyma. The inclusion of additional contours at threshold attenuation measurements for large tumors could be used to show a more specific correlation between areas of enhancement within a tumor and local microsphere uptake. Voxel-based parameters within the volumetric contours may also be helpful in providing a more granular understanding of microsphere distribution within larger tumors. Finally, analysis of studies in which angio-CT images are available from both mapping and treatment could provide additional understanding about whether enhancement features might change between the mapping and treatment, potentially explaining some of the studies with poor correlation between enhancement, SPECT, and PET.

## Conclusions

In this study, the ENR on angio-CT performed as well as ^99m^Tc-MAA in the prediction of ^90^Y PET activity distribution. Comparison of the ENR on planning arteriography prior to ^99m^Tc-MAA injection and during the ^90^Y administration may shed light on conflicting observations in the literature about the utility of ^99m^Tc-MAA in the prediction of ^90^Y activity distribution. This parameter may only be useful currently as an adjunct to standard of care practice which includes ^99m^Tc-MAA administration; however, it could be used independently in ^90^Y dosimetry if validated in future studies and a suitable substitute could be found for determining the lung shunt fraction.
